# Enhanced Auditory Arousal Increases Intake of Less Palatable and Healthier Foods

**DOI:** 10.5539/gjhs.v6n3p1

**Published:** 2014-01-23

**Authors:** Gregory J. Privitera, Melissa Diaz, Meagan C. Haas

**Affiliations:** 1 Department of Psychology, Saint Bonaventure University, New York, USA

**Keywords:** arousal hypothesis, healthy foods, volume, consumption, palatability

## Abstract

Two experiments were conducted to test a prediction of the arousal hypothesis that increased arousal will increase intake of less palatable and healthy foods. In both experiments, arousal was manipulated by adjusting the volume of a movie (soft, loud volume) while participants consumed foods. In Experiment 1, participants ate fresh (palatable) or stale (less palatable) popcorn during a 9-minute movie played at a soft or loud volume. Experiment 2 used the same procedures with healthier foods (carrot sticks and apple slices). Partial support for the arousal hypothesis in Experiment 1 showed that participants consumed more stale but not fresh popcorn in the loud (high arousal) versus soft (low arousal) volume group. These findings suggest that low but not high palatable foods are susceptible to manipulations of arousal. Consistent with this interpretation, Experiment 2 showed that high but not low environmental arousal increased intake of the fruits and vegetables, which are typically rated as lower in palatability compared to high fat foods. These results show that high arousal in an eating-typical environment increases intake of less palatable foods, and healthy foods (i.e., fruits and vegetables). Increasing the availability of healthier foods in a loud food environment can have a positive impact on increasing intake of fruits and vegetables in that environment.

## 1. Introduction

Many studies show that the food environment (a place where food is actually consumed) can substantially influence food intake and consumption volume of food, which can lead to overeating and subsequent obesity (Drewnowski, 2004; Swinburn, Sacks, McPherson, Finegood, Moodie, & Gortmaker, 2011). Because intake of unhealthy foods is associated with rising rates of obesity in the U.S. (Wadden, Brownell, & Foster, 2002), a lot of attention has focused to how environmental factors contribute to food intake and diet (Befort, Nazir, & Perri, 2012; Chang & Christakis, 2002). Particular attention has been focused to the microscale food environment, which is the immediate surroundings within which people consume foods and drinks (Sobal & Wansink, 2007). Features in this environment that can influence the amount of food consumed include the setup of the room itself (Painter, Wansink, & Hieggelke, 2002; Stroebele & de Castro, 2004), the furniture in the room (Privitera, Cooper, & Cosco, 2012), the container in which food is stored or served (Wansink & Kim, 2005), and features of the food itself (Geier, Rozin, & Doros, 2006; Kral & Rolls, 2011; Spiegel, 2000).

An additional feature in a microscale environment that can influence intake is the background noise in that environment. Many people consume foods and drinks in a noisy environment, such as while listening to music or watching TV (Bellisle & Dalix, 2001; Bellisle, Dalix, & Slama, 2004; Blass, Anderson, Kirkorian, Pempek, Price, & Koleini, 2006). To explain these effects, the arousal hypothesis posits that any environmental cue that causes increased arousal will amplify an individual’s behavior towards an external event or object (Smith & Curnow, 1965). Thus, in a microscale food environment this hypothesis predicts that participants will increase intake of foods, possibly even healthier foods, in a loud food environment (high arousal) compared to a lower volume food environment. The food is the external object upon which participants amplify their behavior (i.e., eating).

Studies supporting the arousal hypothesis demonstrate that increased auditory stimulation (Bellisle et al., 2004), and the tempo and rhythm of music (Sloboda, 1991; Van der Zwaag, Westerink, & Broek, 2011) can increase food intake and food choice (Milliman, 1982). Further evidence shows that participants spend more time eating at a restaurant (Caldwell & Hibbert, 2002) and spend more time drinking (Guegen, Jacob, Le Guellec, Morineau, & Lourel, 2008) or drinking at a faster rate (Stafford & Dodd, 2013) in a loud versus low volume environment. However, other researchers report that the time spent eating food and the size of food portions was not impacted by the speed or tempo of music, even when overall intakes were higher in the presence of music (Stroebele & de Castro, 2006). These results suggest that the effects of arousal on food intake may have certain limiting factors to which the arousal effect is restricted.

One possible factor tested here is the type of food consumed in a loud versus a low volume environment. Many studies show that taste alone can control food intake in a variety of settings when the taste itself is highly preferred (Drewnowski, 1997; Mela & Sacchetti, 1991). Therefore, we hypothesized that taste, and not arousal, will control food intake for highly preferred foods, whereas arousal will control food intake when foods are not highly preferred. In an exploratory analysis, we manipulated whether participants consumed fresh (good taste) or stale (poor taste) high-fat popcorn in a loud or low volume setting in Experiment 1. The implications for the findings in Experiment 1 led to the prediction that high arousal will increase intake of healthier foods, such as fruits and vegetables, which according to the Centers for Disease Control and Prevention are underconsumed in the U.S. (Tolhill, 2005). In Experiment 2, we therefore conducted the same study design, except that fruits and vegetables were used to test if high arousal (i.e., loud volume) could increase intake of theses generally less preferred but healthier foods, compared to foods that are high in fat (Drewnowski, 1997; Privitera, 2008).

## 2. Experiment 1

### 2.1 Methods

#### 2.1.1 Participants

A total of 80 participants (44 women, 36 men) were recruited through university classroom visits and sign-up sheets. Participant sample characteristics were (*M*±SD) age (20.3±1.6 years), weight (77.6±8.6 kg), height (176.5±5.6 cm), and BMI (24.9±2.0 kg/m^2^). The 80 participants passed an initial screening used to determine if individuals qualified to participate. Only those who were non-smokers in general good health with no physical or doctor diagnosed food allergies, medical conditions including pregnancy and eating disorders, or dietary restrictions were included in data analyses. Because individuals with a history of dieting can be insensitive to flavor-based learning (Brunstrom, Downes, & Higgs, 2001), all participants scoring 9 or higher on the Restraint Scale of the Three Factor Eating Questionnaire (Stunkard & Messick, 1985) were excluded. Hunger states also influence flavor-based learning (Yeomans & Mobini, 2006; Yeomans, 2006), so participants who ate within two hours of the study were also excluded. In addition, all participants reported having no history of impaired hearing.

#### 2.1.2 Setting and Foods

*Auditorium Setting*. A video clip was presented in an auditorium theater setting for all groups. The auditorium had three sections of seating separated by two stair isles with a large screen projector to the front of the room and surround sound throughout the room. All participants sat in the middle, front two rows where the volume was consistent—the decibels (db) of the movie did not vary within each volume group. The distance from the seating area to the viewing screen was approximately 3.7 m to 6.1 m.

*Movie Clip*. The movie was a commercial-free 9-minute video clip of “Americas Funniest Home Videos.” This movie was used because it did not have a story line, which made it easy to understand without having to follow a story line. The volume of the movie was made soft (low volume; 50 db) or loud (high volume; 80 db) to manipulate arousal. Participants reported that the volume was not aversive or distracting at each decibel level. Only high and low volume (i.e., arousal) groups were included to explore differences in intake between groups with obvious differences in volume.

*Popcorn*. The foods used were 45 grams of fresh or stale (one-week old) Orville Redenbacher’s Movie Theatre Butter popcorn (ConAgra Foods, Inc., Omaha, NE, wt/vol). The stale popcorn was popped and then stored at room temperature in sealed containers one week prior to the experiment. The size of a portion was 45 grams for each participant. “Seconds” were allowed upon request only if the participant consumed the entire first portion. Amount consumed was recorded as the difference in weight (g) of the portion pre-post movie. Spillage was accounted for before recording differences in the weights of the popcorn because the participants were in identifiable locations in the auditorium.

#### 2.1.3 Procedures

Participants were observed in groups of 8 to 10 at a time between 3:00 PM and 5:00 PM EST, were asked to sit in the middle front two rows of the auditorium, and then read and signed an informed consent prior to watching the movie. The db of the movie did not vary in the front two rows so all participants experienced the same db volume (soft or loud, depending on group assignment). Participants were randomly assigned to groups (*n* = 20 per group) based on the volume of the movie (soft, loud) and the freshness of the popcorn served (fresh, stale). Group Soft-Fresh consumed fresh popcorn during a movie played at 50 db. Group Soft-Stale consumed stale popcorn during a movie played at 50 db. Group Loud-Fresh consumed fresh popcorn during a movie played at 80 db. Group Loud-Stale consumed stale popcorn during a movie played at 80 db. Otherwise, all procedures were the same for all groups.

Each participant received the same sized container filled with either fresh or stale popcorn and then were played the movie clip at a soft or loud volume, depending on group assignment. Prior to the movie, participants completed an affect grid (Russell, Weiss, & Mendelsohn, 1989), which is used to record changes in both mood and arousal. To check hunger states, participants also completed a rating scale from 1 = *not hungry at all* to 7 = *very hungry* to the following item: “How hungry are you at this moment?” After the movie, participants completed the same affect grid, and again rated their hunger state, and also the palatability of the popcorn by stating their level of agreement from 1 (*not at all*) to 7 (*very good*) to the following single item: “The popcorn tasted good.” In addition, they completed the Estimated Daily Intake Scale for Fat (EDIS-F; Privitera & Freeman, 2012) to estimate daily intake of fat, which was included as a covariate in statistical analyses because the test food used was a high fat food (65% of calories in the popcorn come from fat). Higher scores on the EDIS-F indicate greater daily intake of fat. Once completed, participants were given a debriefing form, thanked for their time, and dismissed. The university’s Institutional Review Board approved all procedures.

#### 2.1.4 Data Analyses

A two-way between-subjects analysis of covariance (ANCOVA) was computed with movie volume (loud, soft) and popcorn (fresh, stale) as the between-subjects factors, and BMI and EDIS-F scores as the covariates. Gender was included as a factor, but removed when it showed no significance with effects reported here. Hence, all analyses reported here did not vary by gender, and also include statistical control for participant differences in BMI and daily intake of fat. The dependent variables were popcorn intake (g), and ratings of palatability, mood, and arousal. Tukey’s HSD was used as the post hoc test. All tests were conducted at a .05 level of significance.

#### 2.2 Results

**Figure 1:**
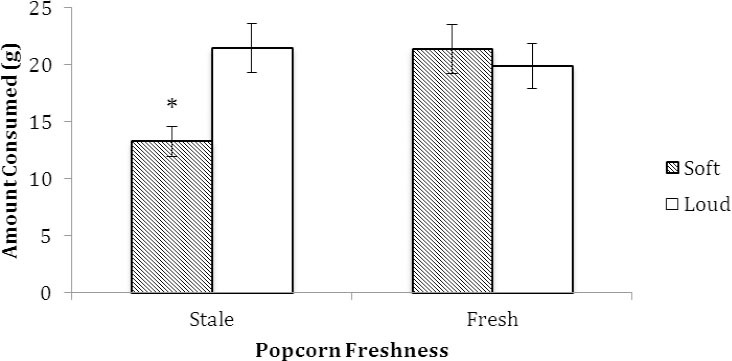
Total amount consumed (g) of fresh and stale popcorn in the soft and loud volume groups in Experiment 1. An asterisk indicates significance at *p* = .01. Vertical bars indicate standard error of the mean (SEM)

As shown in Figure 1, with food intake as the dependent variable, a significant freshness × volume interaction was evident, F(1, 74) = 6.98, *p* = .01 (*R^2^* = .09). Simple main effect tests showed that popcorn intakes did not differ between the soft and loud volume groups when fresh popcorn was consumed (*p* = .50). For participants eating stale popcorn, however, intake was greater in the loud volume (*M* = 21.45, SD = 8.92 grams) versus soft volume (*M* = 13.30, SD = 4.35 grams) group, Tukey’s HSD, *p* < .05.

With palatability as the dependent variable, a significant freshness × volume interaction was evident, F(1, 74) = 6.28, *p* = .014 (*R^2^* = .09). Simple main effect tests showed that palatability ratings did not differ between the soft and loud volume groups when fresh popcorn was consumed (*p* = .43). For participants eating stale popcorn, however, palatability ratings were greater in the loud volume (*M* = 5.60, SD = 1.50) versus soft volume (*M* = 4.20, SD = 1.51) group, Tukey’s HSD, *p* < .05.

Pre-movie mood and arousal scores did not significantly differ between groups (*p* > .40). Post-movie analyses with mood as the dependent variable showed a significant main effect of freshness, F(1, 74) = 6.02, *p* = .016 (*R^2^* = .08), with participants reporting higher mood after eating fresh (*M* = 2.13, SD = 1.62) versus stale popcorn (*M* = 1.23, SD = 1.48). With arousal as the dependent variable, a main effect of volume was significant, F(1, 74) = 4.43, *p* = .039 (*R^2^* = .06), confirming the arousal manipulation, showing that participants reported being more aroused in the loud (*M* = 2.03, SD = 1.94), compared to the soft volume (*M* = 1.08, SD = 1.39) groups. Changes in hunger ratings did not vary by group (*p* > .50).

## 3. Experiment 2

The results in Experiment 1 show only partial support for the arousal hypothesis. Specifically, the arousal hypothesis was supported only for the stale popcorn groups: When popcorn was stale, increased arousal (80 db movie) increased intake and perceived palatability and mood. No effect on intake was observed in the fresh popcorn groups. A possible explanation for this outcome is that when the taste of a food is very good (such as for fresh popcorn), then taste, and not arousal controls food intake. However, when palatability or taste is low, arousal is more salient and therefore has greater control over food intake.

An implication of the outcome in Experiment 1 is that increased arousal should also be able to enhance intake of healthier, typically less palatable foods—compared to high fat, high sugar foods (Privitera, 2008; Privitera, Antonelli, & Creary, 2013). If true, then increased arousal could effectively enhance intake of healthier foods, when these foods are made available during a movie. This possibility was tested in Experiment 2. Specifically, we tested the hypothesis that increased arousal can increase intake of fruits (i.e., apple slices) and vegetables (i.e., carrot sticks) in the same setting as described for Experiment 1.

### 3.1 Method

#### 3.1.1 Participants

A total of 43 participants (23 women, 20 men) were recruited through university classroom visits and sign-up sheets. Participant sample characteristics were (*M*±SD) age (20.6±1.7 years), weight (79.8±7.8 kg), height (175.2±6.4 cm), and BMI (25.0±2.4 kg/m^2^). All participants were selected for Experiment 2 using the same criteria as described for Experiment 1.

#### 3.1.2 Setting and foods

The setting was the same as described in Experiment 1. The foods used in Experiment 2, were sweet red apple slices (Country Fresh, Inc., Houston, TX, wt/vol) and baby-cut carrots (Bolthouse Farms, Inc., Bakersfield, CA, wt/vol). Pre-sliced apples and baby-cut carrots were used to ensure that all cuts of the fruits and vegetables were similar and to avoid bruising of the apples. The foods were approximately 6 calories per slice or cut and allowed for a comparable portion size across both foods.

#### 3.1.3 Procedures

Same as described in Experiment 1, except that participants were given portions of apple slices and carrot sticks simultaneously. Portions were 10 apples slices and 10 carrot sticks each placed in front of a participant in separate small paper bowls. Hence, participants were assigned to one of two groups: Low arousal (soft volume; 50 db; *n* = 22) and high arousal (loud volume; 80 db; *n* = 21). Amount consumed of apple slices and carrot sticks (in g) in each group were recorded. The university’s Institutional Review Board approved all procedures.

#### 3.1.4 Data Analyses

A one-way between-subjects ANCOVA was computed with groups (soft, loud volume) as the between-subjects factor and BMI as the covariate. Intakes of apple slices (g), carrot sticks (g), and total intakes (g), and ratings of mood, arousal, and palatability were the dependent variables. Gender was included as a factor, but removed when it showed no significance with effects reported here. All tests were conducted at a .05 level of significance.

### 3.2 Results

**Figure 2:**
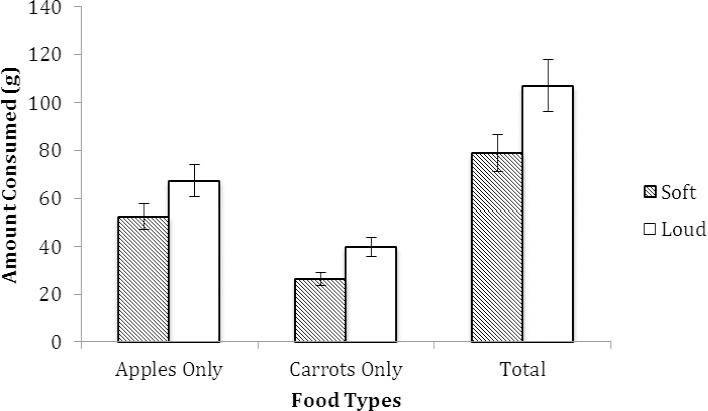
Total amount consumed (g) of apples, carrots, and total intake in the soft and loud volume groups in Experiment 2. For each food type, significantly more food was consumed in the loud vs. soft volume groups. Vertical bars indicate SEM

With food intake as the dependent variable, a significant effect of arousal was evident for total intake, F(1, 39) = 7.42, *p* = .01 (*R^2^* = .20), for intake of apple slices, F(1, 39) = 4.16, *p* < .05 (*R^2^* = .11), and for intake of carrot sticks, F(1, 39) = 4.93, *p* = .03 (*R^2^* = .17). As shown in Figure 2, results show that intakes of apple slices, carrot sticks, and total intakes were higher in the loud volume (high arousal) versus the soft volume (low arousal) group.

Pre-movie mood and arousal scores did not significantly differ between groups (*p* > .55). Post-movie analyses with mood as the dependent variable showed no significant effects of volume, *p* = .60, with mood being similar in the soft and loud volume groups. With arousal as the dependent variable, an effect of volume was significant F(1, 39) = 4.79, *p* = .035 (*R^2^* = .11), confirming the arousal manipulation, showing that participants reported being more aroused in the loud (*M* = 2.91, SD = 2.35), compared to the soft volume group (*M* = 1.19, SD = 2.23). Palatability ratings for apples (*p* = .94) and carrots (*p* = .23) also did not significantly differ between groups. Changes in hunger ratings did not vary by group (*p* > .35).

## 4. General Discussion

The hypothesis that increased arousal will enhance intake of less palatable and healthy foods was tested. In two experiments, participants were given foods to eat during a short movie clip that was played at a soft (low arousal) or loud (high arousal) volume. The results in Experiment 1 showed support for this hypothesis with stale (less palatable) popcorn, but not with fresh popcorn. In Experiment 2, the results showed support for the arousal hypothesis using healthy foods (i.e., carrot sticks and apple slices).

The results in Experiment 1 suggest that when food tastes really good, then taste alone, and not arousal (an environmental factor) controls food intake, as is supported by many studies showing strong preferences for the taste of highly palatable high fat foods (Drewnowski, 1997; Mela & Sacchetti, 1991). Hence, arousal influenced intake of stale, but not fresh popcorn, during the movie clip in Experiment 1 because the stale popcorn was less palatable, setting the occasion for arousal to control food intake. Consistent with this interpretation, Experiment 2 showed that participants consumed more carrot sticks and apple slices in a high versus low arousal group. While fruits and vegetables can taste good, their taste is typically rated lower than the taste of high fat foods (Drewnowski & Greenwood, 1983; Drewnowski, 1997; Privitera, 2008).

The effects of changes in mood and perceived palatability of foods also varied between the experiments. In Experiment 1, a high arousal group showed increased intake of stale popcorn, and also increased positive mood and higher palatability ratings of the food consumed compared to a low arousal group. Based on these results, we can surmise that increased intake of stale popcorn was modified by the arousal, and also modified by the increased mood and perceived palatability of the foods (Drewnowski, 1997; Mela et al., 1991; Privitera, 2008). Hence, we cannot be fully certain that arousal alone caused the increased intake. In Experiment 2, however, only intake differed between the low and high arousal groups—no changes were observed in mood and perceived palatability of the foods. Hence, even in the absence of changes in mood and food palatability, enhanced arousal increased intake of the test foods in Experiment 2 (i.e., carrot sticks and apple slices). The pattern of results observed here demonstrate that arousal was sufficient to increase intake of healthy foods and leads to the prediction that increasing arousal in the food environment will increase intake of less palatable foods to include increased intake of fruits and vegetables, as was specifically shown in Experiment 2.

The immediate surroundings of a food environment include the room itself, the furniture, the food container, and the food itself (Sobal & Wansink, 2007). The setting used in Experiments 1 and 2 had the same immediate surroundings, but varied on the arousal (noise) level in the surroundings. Environmental factors can have a substantial impact on consumption volume and food intake (Drewnowski, 2004; Swinburn et al., 2011), even for healthy foods (Privitera & Creary, 2013). In this study, we show for the first time that people will eat more fruits and vegetables in a highly arousing (i.e., loud) environment, which is a common setting for eating foods (Bellisle & Dalix, 2001; Bellisle et al., 2004; Blass et al., 2006). Considerable data coming from the Centers for Disease Control and Prevention show that fruits and vegetables are underconsumed in the U.S. (Tohill, 2005). Hence, a key implication for the pattern of data demonstrated here is that increasing the availability of healthier foods in a loud microscale food environment (i.e., a common eating setting) may have a positive impact on increasing intake of fruits and vegetables in that environment.

## 5. Limitations and Conclusion

In two experiments, we tested the hypothesis that increased arousal will enhance intake of less palatable and healthy foods. The results showed that increased arousal increased intake of less palatable popcorn (i.e., stale popcorn), and healthier foods, which taste good, but are typically rated as less palatable or less liked than high fat foods such as fresh popcorn. Whether these findings may have been influenced by the type or genre of movie presented, or whether these findings are also likely to occur when people are alone in the room cannot be determined based on the procedures used here. Further studies are needed to better understand the possible mechanisms causing this effect, and to extend the ecological validity of these findings by testing across a variety of eating-typical settings and situations.
